# Metabolomic and gene expression approaches reveal the developmental and environmental regulation of the secondary metabolism of yacón (*Smallanthus sonchifolius*, Asteraceae)

**DOI:** 10.1038/s41598-019-49246-2

**Published:** 2019-09-11

**Authors:** Guillermo F. Padilla-González, Maximilian Frey, Javier Gómez-Zeledón, Fernando B. Da Costa, Otmar Spring

**Affiliations:** 1 AsterBioChem Research Team, Laboratory of Pharmacognosy, School of Pharmaceutical Sciences of Ribeirão Preto, University of São Paulo, Av do café s/n, 14040-903 Ribeirão Preto, SP Brazil; 2 Institute of Botany, University of Hohenheim, Garbenstraße 30, 70599 Stuttgart, Germany

**Keywords:** Metabolomics, Secondary metabolism

## Abstract

Acting as chemical defense or signaling compounds, secondary metabolites (SMs) play an essential role in the evolutionary success of many angiosperm plant families. However, the adaptive advantages that SMs confer, and the influence of environmental and developmental factors on SMs expression, remains poorly understood. A study of taxa endemic to the variable Andean climate, using a metabolomics approach, may provide further insight. By analyzing gene expression patterns and metabolic fingerprints, we report herein the developmental and environmental regulation of the secondary metabolism of *Smallanthus sonchifolius* (yacón), a medicinal Andean plant. Our results demonstrate a clear developmental stage dependent regulation of the secondary metabolism of yacón leaves wherein the metabolic diversity increases with plant age. However, environmental factors seem to regulate biosynthetic pathways, creating differences in the expression of chemical classes, pointing to an association between transcription levels of relevant genes and the relative amounts of more than 40 different metabolites. This study suggests that the secondary metabolism of yacón is regulated by a complex interplay between environmental factors and developmental stage and provides insight into the regulatory factors and adaptive roles of SMs in Andean taxa.

## Introduction

With more than 23,000 species currently described and a global distribution, Asteraceae represents one of the largest angiosperm plant families. Secondary metabolites are partly attributed to its evolutionary success^1–4^. According to Ehrlich and Raven (1964), plant diversification is often the result of innovation in defenses against natural enemies^5^. Therefore, in plant groups with high diversification rates, such as Asteraceae, the evolution of novel chemical defenses is related to a higher ecological success and increased speciation. The high diversity of Asteraceae, especially in the tropical Andes of South America^6^, is reflected in the large array of secondary metabolites that have been identified in some of its members^4^. Flavonoids, *trans*-cinnamic acid derivatives, polyacetylenes, coumarins and sesquiterpene lactones (STLs), in addition to other terpenoids and acetylenes, constitute characteristic metabolites commonly found in Asteraceae^4^. These metabolites confer well-known ecological roles, such as pollinator attraction, allelopathy, and protection against parasites, pathogens, herbivores, and abiotic environmental factors^7^. Therefore, secondary metabolites constitute the chemical interface between the plant and its environment, an interaction mediated by potentially thousands of chemical species.

It is well documented that climatic factors such as water availability, temperature and solar radiation can influence the biosynthesis of metabolites from different chemical classes^8–10^. An example of this is the substantially raised levels of phenolic compounds accumulated in the upper epidermal layer of plant cells in response to high levels of UV-B radiation^11,12^. Furthermore, the presence of water-soluble polymers of fructose, such as fructans and inulins in some species in Asteraceae, e.g., *Smallanthus sonchifolius* (Poepp. & Endl.) H. Robinson, is another example, as these molecules are associated with the plant’s ability to withstand low temperatures and drought conditions^13,14^. The accumulation of these fructose polymers is hypothesized to be behind the adaptive success of the family in the seasonally dry and cold environments characteristic of some tropical Andean ecosystems^15^. The capitate glandular trichomes present in aerial parts of many Asteraceae species is a clear example of resistance to herbivores^16^. High amounts of STLs are biosynthesized and stored in this type of specialized trichomes^17–19^, acting as herbivore-deterrents^20^ and serving as allelochemicals^21^. However, in spite of the evidence to corroborate such adaptive roles for secondary metabolites in the family Asteraceae, most studies monitor single compounds or chemical classes under controlled laboratory conditions. Therefore, the environmental and developmental factors influencing the secondary metabolism of Andean taxa remains poorly understood.

*Smallanthus sonchifolius*, popularly known as yacón, is a perennial herb native to the South American Andes^22^. Yacón is a fast-growing species with a high adaptive plasticity to diverse environments. This species is currently cultivated in numerous countries of Europe and Asia for its medicinal^23,24^ and nutritious properties^25^. Given its remarkable adaptive success in diverse environments and rapid growth, yacón can be considered as a model organism to use in elucidating the influence of intrinsic (e.g., plant developmental stage) and extrinsic environmental factors on the biosynthesis and accumulation of secondary metabolites in Andean groups. Recent studies demonstrate that the secondary metabolite chemistry of *S*. *sonchifolius* is characterized by the presence of flavonoids, caffeic acid derivatives (CADs), diterpenes and STLs^23,26–29^. As a first step in understanding the environmental and developmental regulatory factors and adaptive roles played by secondary metabolites in Andean groups, we aimed to correlate the metabolome of yacón leaves with gene expression patterns against environmental variables and developmental stages.

**Figure 1:**
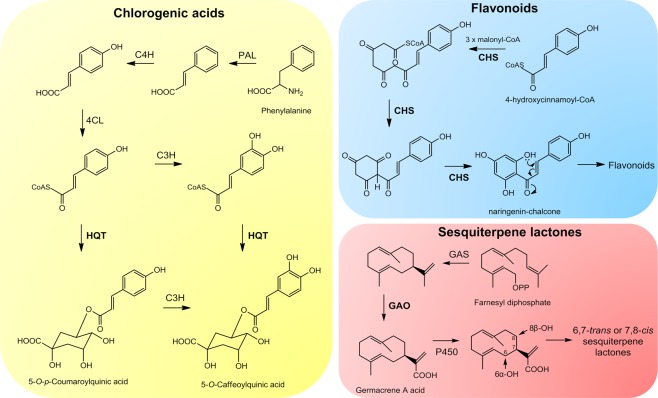
Biosynthetic pathways of chlorogenic acids, flavonoids and sesquiterpene lactones in Asteraceae. Chlorogenic acid enzymes: phenylalanine ammonia lyase (PAL), cinnamate 4′-hydroxylase (C4H), 4-cinnamoyl CoA ligase (4CL), hydroxycinnamoyl CoA: quinate hydroxycinnamoyl transferase (HQT), *p*-coumaroyl-3′-hydroxylase (C3H); flavonoid enzymes: chalcone synthase (CHS); sesquiterpene lactone enzymes: germacrene A synthase (GAS), germacrene A oxidase (GAO).

Metabolomics, which is defined as the study of the complete set of metabolites synthesized by an organism in response to genetic or environmental changes, has recently emerged–aiming to provide a link between genotypes and phenotypes^30^. By using modern analytical platforms and computational methods, this relatively new research field has promoted a broader analysis of plant metabolism, leading to a better understanding of the interactions of organisms with their environments^31^. In this context, metabolomics constitutes an interesting approach to the study of the influence of abiotic environmental factors on the metabolism of higher plants^10^, including Andean taxa. The possibility of monitoring the metabolome of yacón leaves in different environments and developmental stages, and cross examining against the expression levels of key genes, could greatly improve the understanding of the adaptation mechanisms of Andean plants. Additionally, considering that yacón constitutes a functional food and medicinal plant commercialized for the treatment of hyperglycemic disorders, among which STLs and CADs play an active role^32,33^, the identification of the environmental or developmental regulatory factors modulating the biosynthesis of these metabolites can benefit in the development of reliable commercial products. Thus, given the ecological significance and medicinal importance of chlorogenic acids (CGAs), flavonoids and STLs, their expression levels were monitored, at different developmental stages, using key genes involved in their biosynthetic pathway; (hydroxycinnamoyl CoA: quinate hydroxycinnamoyl transferase (HQT), chalcone synthase (CHS) and germacrene A oxidase (GAO), respectively (Fig. 1).

## Results

### Metabolic fingerprinting and correlation with developmental and environmental data

**Figure 2:**
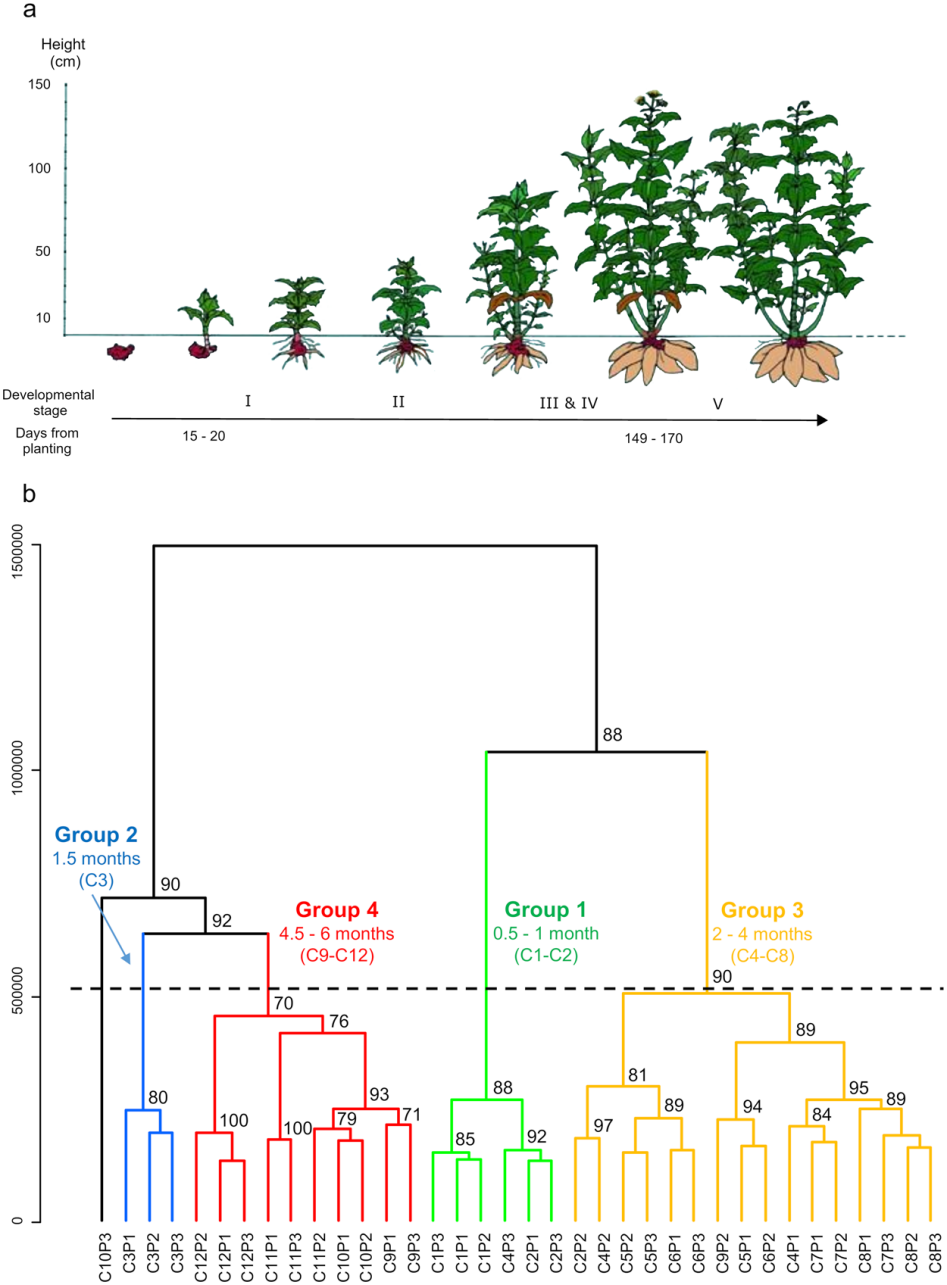
(**a**) Graphical representation of the developmental stages of yacón (image by Prof. Dr. Eloy Fernandez, Czech University of Life Sciences) and (**b**) HCAbp based on metabolic fingerprinting by UHPLC-UV-HRMS in positive mode of 36 extracts from *Smallanthus sonchifolius* leaves collected along the development of the plant (C1 to C12). Proposed groups of samples with similar metabolic fingerprints are highlighted in different colors.

In conducting metabolic fingerprinting via ultra-high-performance liquid chromatography coupled to UV and high-resolution mass spectrometry (UHPLC-UV-HRMS) of yacón leaves collected in different developmental stages (Fig. 2a), 1,562 and 1,353 mass signals were recorded in the positive and negative ionization modes, respectively. Based on Hierarchical Clustering Analyses with bootstrap resampling (HCAbp), the positive ionization mode dataset was chosen for further analysis. This mode was selected as a basis for proposing different groups of samples with similar metabolic fingerprints (Fig. 2b).

Results from HCAbp (Fig. 2b) showed a clear clustering tendency by developmental stage in the metabolic fingerprints of yacón leaves, with four main groups. Group 1 clusters the first two collections (C1 and C2), corresponding to leaves produced by 15 and 30-day-old plants, respectively, while leaves from 1.5-month-old plants formed an independent cluster in the HCApb (Group 2, Fig. 2b). Similarly, leaves from two to four-month-old plants clustered in an independent group (Group 3, Fig. 2b), while leaves from the last stages of plant development (between 4.5 and six-month-old plants) clustered in group 4 (Fig. 2b). Interestingly, leaves from 1.5-month-old plants (Group 2, Fig. 2b) appear to be metabolically more similar to the leaves produced by plants reaching the adult stage (Group 4, Fig. 2b) than to the leaves produced by slightly younger or older plants. Additionally, different subclusters segregate specific collections in groups 3 and 4 (Fig. 2b). For example, collections 7 and 8, corresponding to 3.5 and four-month-old plants (Fig. 2b), tend to form an independent subcluster in group 3, with a similar but less pronounced pattern observed among collections 9 and 10 in group 4 (4.5 and five-month-old plants, respectively, Fig. 2b). In most cases, high bootstrap values (>80%) were obtained, indicating good support for the obtained clusters.

**Figure 3:**
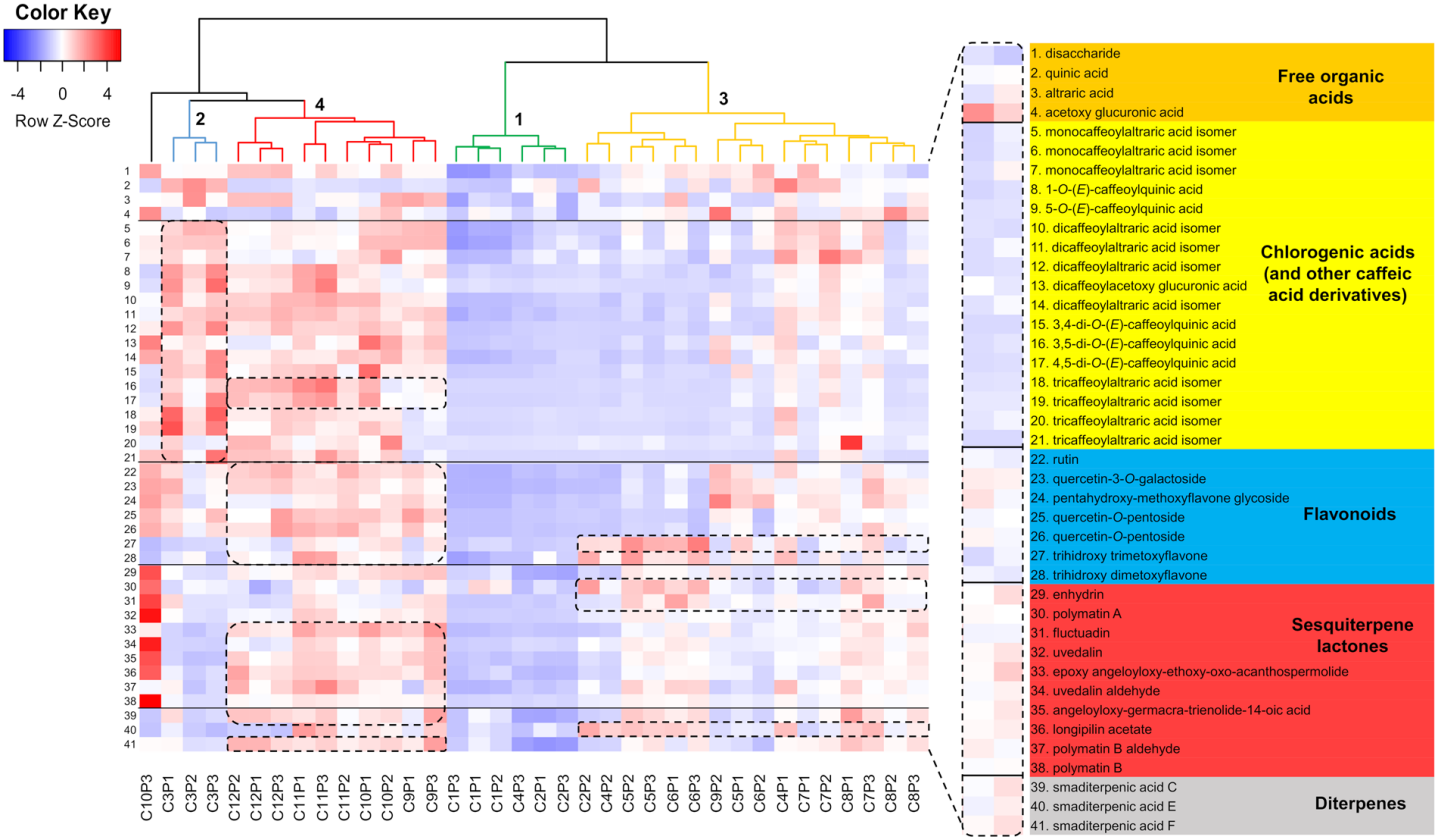
Heatmap showing the differential accumulation of 41 metabolites in 36 extracts from *Smallanthus sonchifolius* analyzed by UHPLC-UV-HRMS. Dash boxes highlight the preferential accumulation of metabolites by the four different groups of samples.

Further analysis of the same dataset by Orthogonal Partial Least Squares-Discriminant Analysis (OPLS-DA) (R^2^ = 0.93, Q^2^ = 0.74, Supplementary Fig. S1) and heatmaps (Fig. 3) pointed to the discriminant metabolites responsible for the observed clustering tendency in the HCAbp. Leaves from 1.5-month-old plants (Group 2, Fig. 3) accumulate mostly CGAs and other caffeic acid derivatives, of which 1-*O*-caffeoylquinic acid is the major discriminatory compound for this group, along with its isomer 5-*O*-caffeoylquinic acid (Fig. 3). CGAs are also accumulated in high amounts (although to a lesser extent) by samples belonging to group 4 (Fig. 3), corresponding to 4.5 and six-month-old plants, explaining the close clustering distance exhibited among samples in groups 2 and 4 (Figs 2b and 3). However, samples in group 4 are easily distinguished from group 2 by the accumulation of STLs and diterpenes (Fig. 3). The STL enhydrin and the diterpene smaditerpenic acid F appear as the main discriminatory substances of group 4 based on the OPLS-DA model. The accumulation of several metabolites from different chemical classes within this group of nearly adult and actual adult plants (Group 4, Fig. 3) suggests that plant age affects the metabolism of yacón, as adult plants are metabolically more diverse than their younger counterparts. The low chemical diversity found among 15 and 30-day-old plants further supports this fact (Group 1, Fig. 3), as the clustering tendency of samples belonging to this group is rather due to their poor metabolic diversity (Fig. 3). Leaves collected from two to four-month-old plants (Group 3, Fig. 3) accumulate intermediate amounts of CGAs, flavonoids and STLs, although some metabolites such as smaditerpenic acid E, trihydroxy trimethoxyflavone, polymatin A and fluctuadin are accumulated in high amounts (Fig. 3).

**Figure 4:**
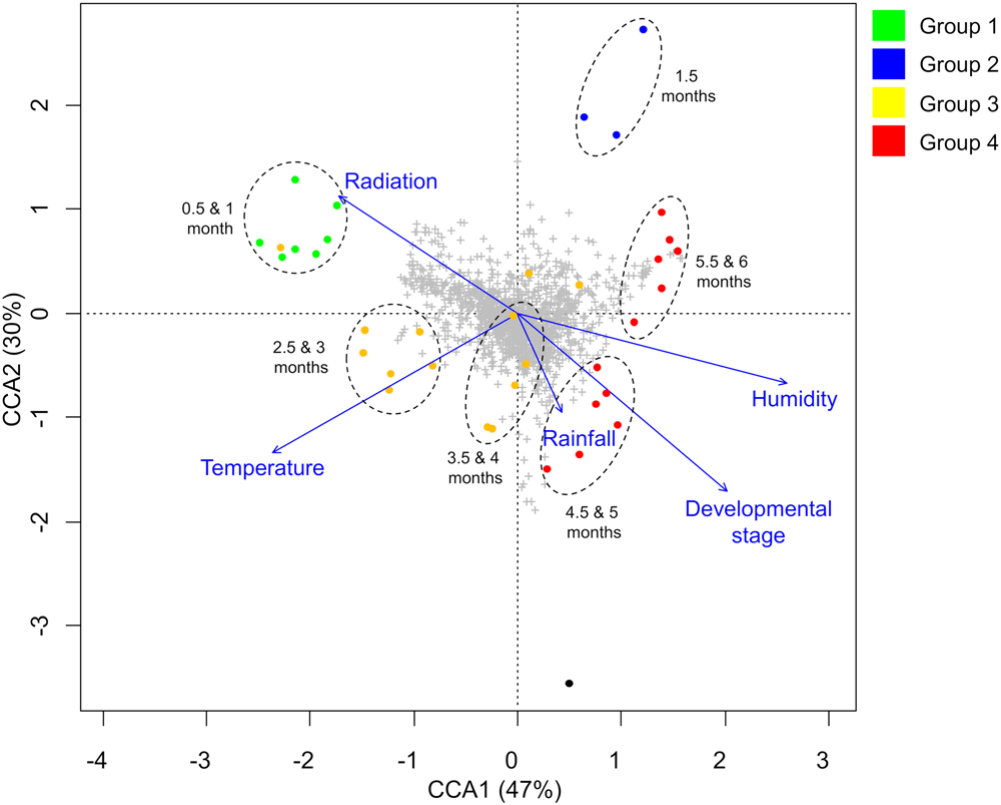
CCA based on metabolic fingerprinting by UHPLC-UV-HRMS in positive mode of 36 extracts from *Smallanthus sonchifolius*, showing the correlation of environmental factors and developmental stage with the clustering of samples by metabolic similarity.

Results from Canonical Correspondence Analysis (CCA) (Fig. 4) and climatograms (Supplementary Fig. S2) allowed for correlating environmental variables and the plant developmental stage at the time of collection with the samples’ metabolic fingerprints. This analysis revealed that each of the environmental factors considered had a different impact on the metabolic fingerprints of yacón leaves. Intermediate to high levels of solar radiation and temperature, in addition to early developmental stages and humidity, seem to influence the metabolic fingerprints displayed by yacón leaves from 0.5 and one-month-old plants (Group 1, Fig. 4). The independent clustering of yacón leaves from 1.5-month-old plants (Group 2, Fig. 4) is correlated with the highest levels of solar radiation (Supplementary Fig. S2) and low temperatures (Fig. 4). On the other hand, the metabolic fingerprint displayed by yacón leaves from a subcluster in group 4, containing 5.5 and six-month-old plants, is associated to high humidity, in addition to low temperatures and low levels of solar radiation (Fig. 4). A similar pattern is observed at the other subcluster in group 4, containing 4.5 and five-month-old plants, but this group is also correlated to high temperatures (Fig. 4) in addition to high humidity and developmental stage. The metabolic fingerprints displayed by 2.5 and three-month-old plants are correlated with high temperatures and intermediate levels of solar radiation and humidity, while in 3.5 and four-month-old plants, high precipitation and high temperatures seem to be the two most important factors determining their metabolic composition (Fig. 4).

### Combined metabolomic and gene expression patterns

**Figure 5:**
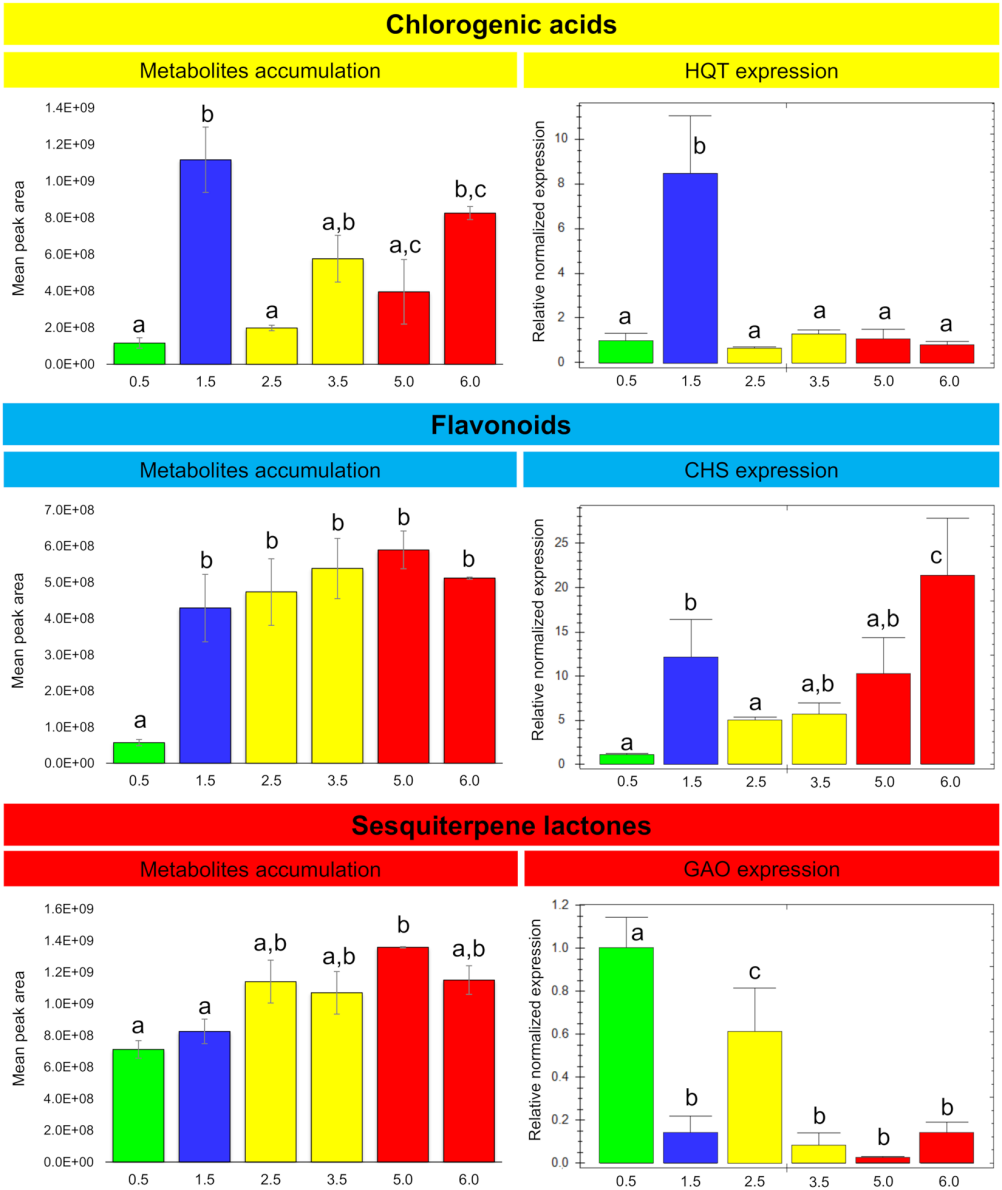
Accumulation of chlorogenic acids (8, 9, 15–17), flavonoids (22–28) and sesquiterpene lactones (29–38) and gene expression of HQT, CHS and GAO during yacón development. Leaf samples collected from 0.5 to 6-months-old plants. Metabolite identities reported in Fig. 3. Normalized gene expression is given relative to the youngest stage and housekeeping genes ACT and EF (n = 3). Error bar: SEM. HQT: hydroxycinnamoyl CoA: quinate hydroxycinnamoyl transferase; CHS: chalcone synthase; GAO: germacrene A oxidase; ACT: actin; EF: elongation factor 1a. Bars colored according to the HCAbp groups (Group 1: green; group 2: blue; group 3: yellow and group 4: red). Same letters into the bar graphs do not differ statistically.

We identified the homologous genes of three key enzymes involved in the biosynthesis of CGAs, flavonoids and STLs in yacón. Blast searches revealed high nucleotide identity to the corresponding genes of closely related Asteraceae: 91% for HQT (relative to *Helianthus annuus* L. HQT), 89% for CHS (relative to *Ageratina adenophora* (Spreng.) R.M. King & H. Rob. CHS) and 85% for GAO (relative to *H*. *annuus* GAO). Although gene expression analyses were carried out for only one gene of each biosynthetic pathway, the selected genes represent key steps in each pathway. Among CGAs, the overall pattern in metabolite accumulation is similar to the HQT expression results (Fig. 5). There is a statistically significant peak in the accumulation of CGAs in 1.5-month-old plants, which is closely mirrored by the gene expression results (Fig. 5). Thus, the accumulation of CGAs in leaves of 1.5-month-old yacón plants is closely associated with the levels of HQT expression. However, after reaching a peak in 1.5-month-old plants, the accumulation of CGAs decreases in the subsequent collections to ultimately increase in the final stages of plant development (Fig. 5), while HQT expression remains low even in the final stages of plant development (Fig. 5). This suggests that the high amounts of CGAs present in adult yacón plants are not associated with higher levels of HQT expression. Analogous to CGAs, the accumulation of flavonoids is proportional to the levels of CHS expression in leaves of 1.5-month-old yacón plants, showing similar patterns in gene expression and metabolite accumulation (Fig. 5). The accumulation of flavonoids increases significantly when plants reach 1.5 months old (Fig. 5); however, contrary to CGAs, no significant changes occur thereafter. Gene expression data for CHS also shows a significant upregulation at 1.5 months (Fig. 5), followed by a significant decrease in the next collection, no significant changes in the following two collections and a significant upregulation at six months (Fig. 5). On the other hand, the accumulation of STLs is inversely proportional to the levels of GAO expression in yacón leaves (Fig. 5). The accumulation of STLs starts early in plant development when plants are 15 days old (or probably even before) and remains stable thereafter (Fig. 5). Gene expression results show an inverse tendency with a significant GAO expression when plants are 15 days old and a subsequent decrease to the final stages of plant development (Fig. 5).

### Molecular networking-based dereplication

Molecular networking was used as a dereplication strategy with the aim of exploring the chemical diversity of yacón and identifying known metabolites and their putative structural analogues in the studied samples^34^. Clustering of the MS/MS spectra based on cosine similarity (>0.65) formed molecular networks with 823 and 491 parent ions visualized as nodes in the positive and negative ionization modes, respectively (Supplementary Figs S3 and S4). Visual inspection of the molecular networks from both ionization modes showed that compounds from the same chemical class tend to cluster together in the network, where seven different chemical classes were identified: caffeoylaltraric acids and derivatives, free glycosides, glycosylated flavonoids, caffeoylquinic acids, STLs, glycolipids and diterpenes (Supplementary Figs S3 and S4).

**Figure 6:**
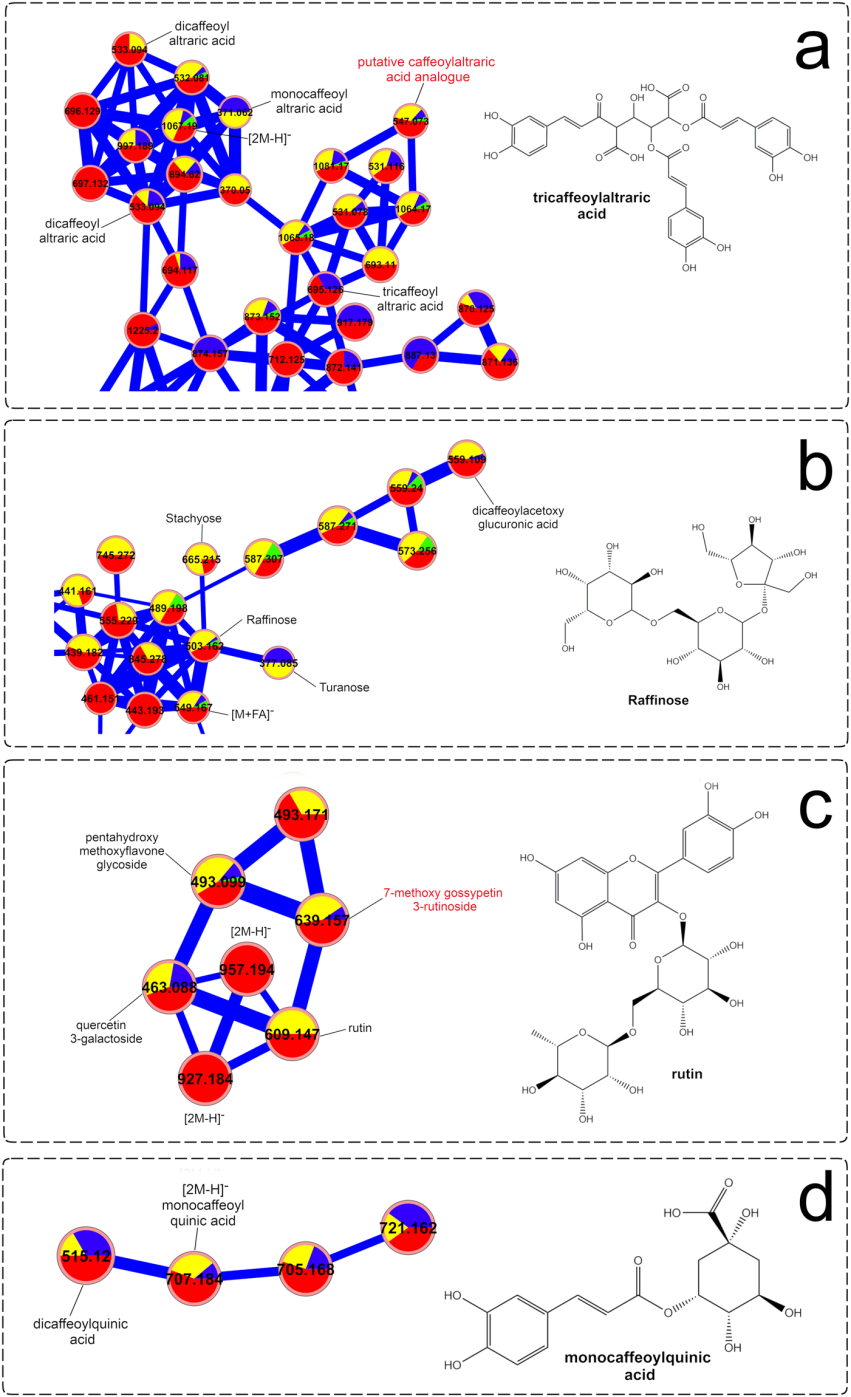
Molecular networks of *Smallanthus sonchifolius*’ leaf extracts (analyzed by UHPLC-HRMS/MS in negative mode) showing specific clusters annotated as (**a**) caffeoylaltraric acids and derivatives, (**b**) free glycosides, (**c**) glycosylated flavonoids and (**d**) chlorogenic acids. The node size represents the mean retention time value of each parent ion with larger nodes representing higher retention times. Node color represents the metabolic developmental stage of the collected samples as follows: green: 0.5 to 1-month-old plants; blue: 1.5-month-old plants; yellow: 2 to 4-month-old plants and red: 4.5 to 6-month-old plants. Edge width depicts the cosine similarity between different MS/MS spectra (0.65 < r > 0.95) with thicker edges representing higher cosine scores.

Analysis of the molecular network from the negative ionization mode (Fig. 6) showed that caffeoylaltraric acids and derivatives represent a chemical class clustering the highest number of nodes, suggesting a high chemical diversity for these molecules in the studied samples. This chemical class was found to be primarily accumulated in 4.5 to 6-month-old plants (Group 4), representing nearly adult and adult plants, and confirming the accumulation patterns seen in the heatmap (Fig. 3). In addition to the mono-, di- and tri-caffeoylaltraric acids previously reported in yacón, other nodes representing putative analogues were also observed, such as the molecule with the *m/z* value of 547.073 (Fig. 6), whose fragmentation pattern is characterized by a neutral loss of 162.032 Da representing the putative loss of a caffeoyl moiety and a base peak at 209.029 *m/z*, representing an ionized molecule of altraric acid. However, the isolation and structural elucidation of this molecule is still necessary to unambiguously determine whether it is a novel metabolite. Interestingly, detailed analysis of the nodes and MS/MS data of the entire network suggest that other cyclic and open chain polyols (in addition to altraric and quinic acid) could also be esterified with caffeic acid molecules at different positions. The putative identification of dicaffeoylacetoxy glucuronic acid (Fig. 6) further supports this hypothesis.

Analysis of the network clusters representing glycosylated flavonoids and CGAs (Fig. 6) allowed the identification of similar trends to the previous analysis by heatmaps (Fig. 3). Here, glycosylated flavonoids are more commonly found among groups 3 and 4 (Fig. 6), corresponding to two to six-month-old plants, while CGAs are found especially in groups 2 (1.5-month-old plants) and 4 (4.5 to six-month-old plants). Among the glycosylated flavonoids (Fig. 6), the clustering of a previously unidentified molecule with a *m/z* value of 639.157, near the nodes corresponding to rutin and quercetin 3-*O*-galactoside (Fig. 6), allowed the proposal of the identity of this molecule as 7-methoxy gossypetin 3-*O*-rutinoside. The fragmentation pattern of this molecule was characterized by a neutral loss of 308.112 Da, suggesting the putative loss of the disaccharide rutinose, and an intense peak at 331.046 *m/z* representing an ionized molecule of 7-methoxy gossypetin. This compound represents a putative new report for the genus *Smallanthus*.

**Figure 7:**
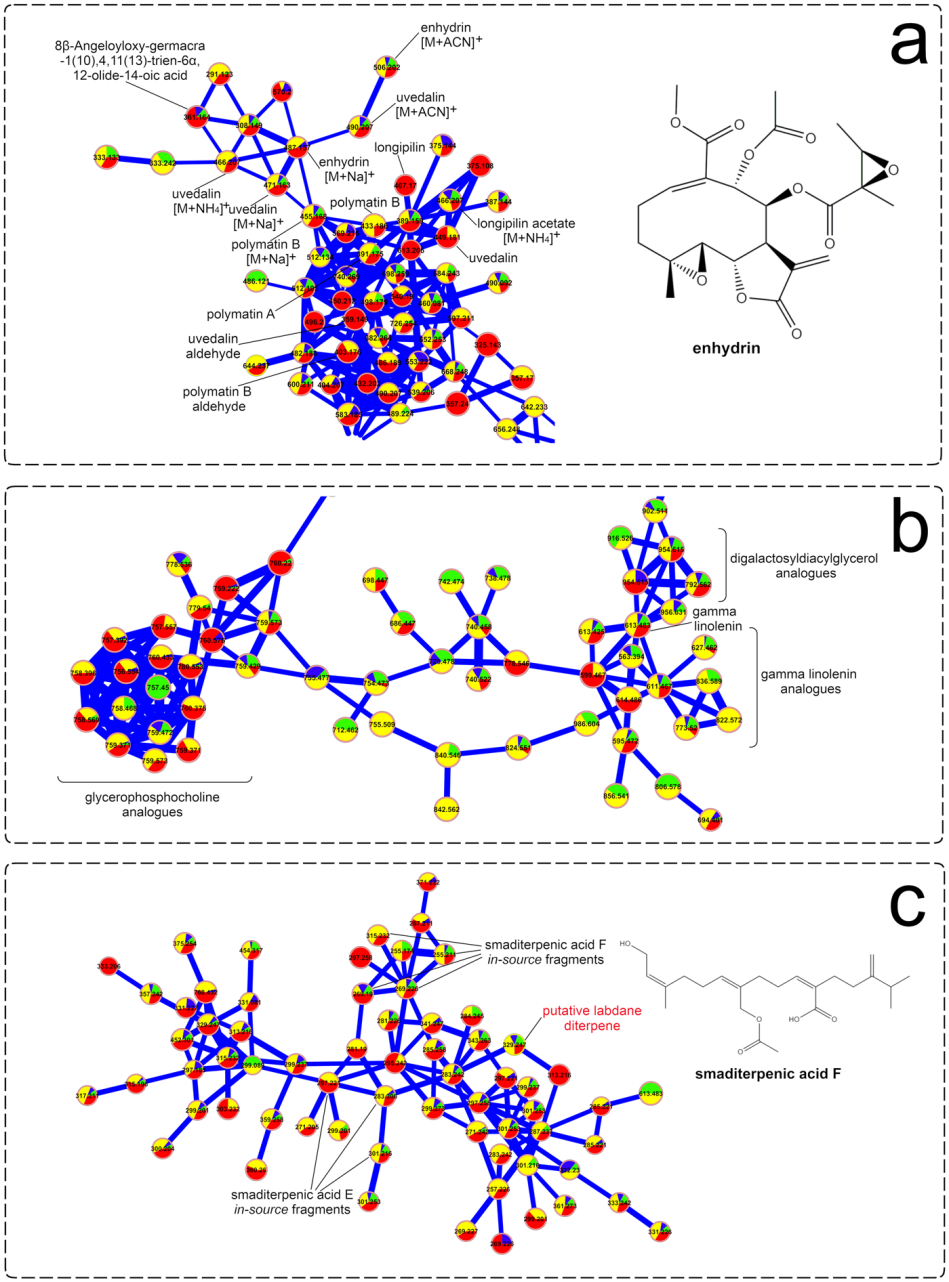
Molecular networks of *Smallanthus sonchifolius*’ leaf extracts (analyzed by UHPLC-HRMS/MS in positive mode) showing specific clusters annotated as (**a**) sesquiterpene lactones, (**b**) glycolipids and (**c**) diterpenes. The node size represents the mean retention time value of each parent ion with larger nodes representing higher retention times. Node color represents the metabolic developmental stage of the collected samples as follows: green: 0.5 to 1-month-old plants; blue: 1.5-month-old plants; yellow: 2 to 4-month-old plants and red: 4.5 to 6-month-old plants. Edge width depicts the cosine similarity between different MS/MS spectra (0.65 < r > 0.95) with thicker edges representing higher cosine scores.

Visual inspection of the molecular network resulting from the analysis of the positive ionization data (Fig. 7) allowed the identification of several STLs previously reported in yacón, in addition to some putative glycolipid analogues and diterpenes based on spectral matches with the GNPS library and detailed manual inspection of the MS/MS data. Among STLs, different adducts of enhydrin and uvedalin, among others (Fig. 7), were identified in all the studied samples, confirming previous results suggesting that the biosynthesis of this chemical class starts early during yacón development and that its presence remains stable through time. In addition to the well-described yacón STLs identified in the present study, molecular networks suggest the putative presence of other STL analogues (Fig. 7), based on their similar fragmentation pattern, which requires further investigation as none of them appear to be reported in yacón to date. Analysis of the diterpene cluster (Fig. 7) allowed the identification of several *in-*source fragments of two well-known yacón diterpenes, namely, smaditerpenic acids F and E, in addition to a putative labdane diterpene based on its similar fragmentation pattern to the labdanes deported in the GNPS library.

## Discussion

This study is a pioneer in reporting the developmental and environmental regulation of the secondary metabolism in a native Andean plant by analyzing gene expression patterns and metabolic fingerprints in an integrated approach. We found that metabolic differences in leaves of yacón are related to developmental and environmental factors and that different classes of secondary metabolites respond differently to such factors. Our results suggest that high levels of solar radiation are associated with the biosynthesis of flavonoids and CGAs in young yacón leaves (1.5-month-old plants), which is in accordance with the literature^10,11,35–39^. These two classes of phenolic compounds (flavonoids and CGAs) are well known to act as protectants of internal tissues against UV-induced damage by absorbing or dissipating solar energy and neutralizing radiation-induced free radicals and other reactive oxygen species^40–42^.

In yacón, it seems that during the earliest stages of plant development (between 0.5 and one-month-old plants) the biosynthesis and accumulation of flavonoids and CGAs is rather low, but apparently once the young yacón plant is exposed to high levels of solar radiation (in addition to low temperatures) there is a significant upregulation in the expression of CHS and HQT, followed by an increase in the accumulation of flavonoids and CGAs, respectively. This suggests that flavonoids and CGAs might also act as UV protectants in young yacón leaves. Previous studies have shown that an increased accumulation of CGAs and flavonoids is associated with increased UV-protection^35–39^. However, the fact that CHS expression and the accumulation of flavonoids increase over time suggests that the environment might not be the only regulatory factor influencing the biosynthesis and accumulation of this chemical class in yacón leaves and that the plant developmental stage is also an important regulatory factor. Samples collected from adult plants at low levels of solar radiation and low temperatures showed comparable amounts of flavonoids and higher CHS expression when compared to their younger counterparts collected at high levels of solar radiation.

On the other hand, it seems that solar radiation constitutes the main regulatory factor influencing the biosynthesis of CGAs (especially of 5-*O*-caffeoylquinic acid) in yacón leaves. Previous studies have shown that 5-*O*-caffeoylquinic acid protects tomato (*Solanum lycopersicum* L., Solanaceae), artichoke (*Cynara cardunculus* L., Asteraceae) and chamomile (*Matricaria chamomilla* L., Asteraceae) against UV-induced damage^37–39^. In the present study, the highest HQT expression and accumulation of CGAs was observed in 1.5-month-old yacón plants which were collected at the highest level of solar radiation. However, a sudden decrease was observed in the concentration of CGAs after 1.5 months. Then, at 2.5-months, the concentration of CGAs was approximately one order of magnitude lower than in 1.5-month-old plants. This statistically significant decrease in the concentration of CGAs suggest that metabolites can be metabolized into storage forms like glycosyl derivatives or broken down to their corresponding forming units (quinic acid and caffeic acid) to be further metabolized into more complex molecules such as lignin. Considering that no glycosyl derivatives were identified in the present study, a possible explanation would be that CGAs are further metabolized into lignin in 2.5-months-old yacón plants. According to Taylor and Zucker (1966), CGAs show a constant turnover in different plant tissues and the turnover of these components has been related to their role as intermediates in the formation of lignin polymers^43^. The fact the stem ramification of yacón (corresponding to the second growth stage) starts between two and 2.5-months after planting, further supports this hypothesis as the stems represent a highly lignified tissue in this species.

Contrary to the observed trend in 1.5 months-old plants, the high concentration of CGAs found in nearly adult and adult individuals are not associated with high levels of HQT expression. The fact that other enzymes, such as hydroxycinnamoyl-CoA:shikimate hydroxycinnamoyl transferase (HCT), and alternative pathways are also capable of converting 4-coumaroyl-CoA to CGA represents a possible explanation for this contrasting trend^44,45^. According to Bartley *et al*. (2016), there are still some uncertainties with respect to the exact nature of the final steps of CGA biosynthesis^46^, as a number of possible routes and enzymes exist to convert 4-coumaroyl-CoA to CGA^47^. For example, HQT and HCT constitute similar enzymes with different substrate preferences: HQT prefers quinic acid, while HCT prefers shikimic acid. However, HCT is still capable of producing 3,5-*O*-dicaffeoylquinic acid, indicating a certain amount of flexibility in substrate specificity^45^. An alternative route in which caffeoyl-*D*-glucose is converted to CGA through hydroxyl cinnamoyl *D*-glucose:quinate hydroxycinnamoyl transferase (HCGQT) has also been reported^44^. Thus, it is possible that other enzymes or alternative pathways could be acting in the later stages of yacón development, explaining the contrasting trend observed in the accumulation of CGAs and HQT expression in adult yacón plants. However, detailed analyses are still necessary to test these hypotheses, as additional factors such as the transcription factor AN1^44^, the content of sucrose^44^ or the turnover mechanism between the biosynthesis of CGA and lignin^43^ could also be involved.

Interestingly, a previous study with carrot (*Daucus carota* L., Apiaceae) roots exposed to UVB showed a linear increase in the accumulation of CGAs over a seven-day study period^46^. At day seven, the average concentration of CGA was six times greater than the concentration found in untreated samples, while the levels of HQT expression in the UVB treatment increased steadily to peak on day three (at approximately 9-fold) and decreased on day six (to 3.3-fold)^46^. This contrasting trend in the accumulation of CGA and HQT expression after an initial peak is similar to the observed trend in our study, therefore indicating that a similar, yet unknown, mechanism must be acting on both carrot and yacón.

Contrary to the observed trends for flavonoids and CGAs, the biosynthesis and accumulation of STLs showed an inverse tendency. The highest GAO expression was observed in 15-day-old plants, while the accumulation of STLs showed a rather stable presence (with a slight increase) over time. A possible explanation for this might be attributed to the age of the leaves. The biosynthesis of STLs in Asteraceae is strongly associated with the leaf developmental stage, with higher expression in leaf primordia and young leaves and subsequent accumulation in capitate glandular trichomes^18,19,48,49^. Although in the present study we always collected the third leaf (from top to bottom), leaf age was not closely monitored. Thus, it is possible that the highest GAO expression seen in 15-day-old plants is due to the analysis of slightly younger leaves relative to the other collections. In yacón, a slow growth occurs during the early stages of plant development^50^, and in the first collection, leaves could be still in a secretory stage, characterized by high levels of gene expression. In the following collections, the secretory activity could have already concluded and STLs were possibly already stored in capitate glandular trichomes, explaining the inverse tendency observed among GAO expression and STLs accumulation. The relatively constant presence of STLs throughout the development of yacón, and their compartmentalization in capitate glandular trichomes, supports a possible protective role for these molecules in the aerial parts of yacón, as the highly nonspecific toxicity of such compounds necessitates compartmentalization to prevent autotoxicity and to protect the plant against herbivores^51^. For instance, STLs are likely to be in part attributable to the evolutionary success of Asteraceae^52^.

Given its high adaptability and plastic response to changing environmental conditions, yacón constitutes a good model organism to understand the adaptive roles of the secondary metabolites that act as a chemical interface between the plant and its environment. Our results suggest that, although plant age seems to strongly influence the metabolism of yacón, it appears that environmental factors such as solar radiation induce a significant upregulation in CHS and HQT expression and in the accumulation of flavonoids and CGAs in young individuals. The accumulation of CGAs can even exceed the extent observed in adult individuals. In yacón, adult plants are metabolically richer and more diverse than their young counterparts are, as demonstrated by the higher number of nodes present in adult plants in the metabolic networks (Figs 6 and 7) and the higher accumulation of metabolites seen in the heatmap (Fig. 3). On the other hand, the early stages of plant development are characterized by a rather poor secondary metabolism. Plants up to one-month old showed the lowest levels of metabolite accumulation and gene expression overall (except for GAO), while intermediate levels are observed in two to four-month-old plants, and the highest levels in metabolite accumulation are usually found in adult or nearly adult individuals. Altogether, our results suggest that in young yacón plants the biosynthesis and accumulation of CGAs and flavonoids is mostly regulated by external environmental factors (e.g., solar radiation), and as the plant grows older, the environment has an attenuated effect on the biosynthesis of both chemical classes. Although CGAs and flavonoids might be both involved in UV protection in yacón leaves, the biosynthesis of each chemical class seems to respond differently relative to the plant developmental stage. CGAs seem to be preferentially biosynthesized and accumulated in 1.5-months-old plants, while the highest concentration of flavonoids and CHS expression was found in nearly adult plants. On the other hand, the concentration of STLs appears to be relatively constant along the development of yacón, with high levels of GAO expression in the early stages of plant development and subsequent accumulation in capitate glandular trichomes. Therefore, our results demonstrate that the secondary metabolism of yacón is regulated by a complex interplay between both plant development and abiotic environmental conditions.

In conclusion, this study reports the developmental and environmental regulation of the secondary metabolism in a medicinally and economically important Andean crop of which scarce information regarding genomic and molecular traits is available. Our comprehensive study of the secondary metabolism of yacón leaves not only allowed the identification of several compounds previously unreported in this species (Supplementary Table S1) but also improves the understanding of the environmental and developmental regulatory factors and possible adaptive roles of different chemical classes of secondary metabolites under changing environmental conditions in Andean taxa. Furthermore, mass spectrometry-based molecular networking allowed the visualization of the chemical diversity of yacón and the detection of several metabolites already described in this species, in addition to some putative novel structural analogues. Lastly, the sequences of the CHS, HQT and GAO genes reported herein provide the first characterization of the putative genes involved in the biosynthesis of flavonoids, CGAs and STLs, respectively, in the genus *Smallanthus*. This finding might effectively encourage the discovery of new genes involved in the secondary metabolism of yacón and its closely related taxa in the future. However, the functional characterization of such genes is still necessary to unambiguously determine their biosynthetic capabilities in model organisms.

## Methods

### Plant material

*Smallanthus sonchifolius* plants were cultivated on a trial field in the Institute of Botany at the University of Hohenheim, Stuttgart, Germany, from April to October 2017 (for 176 days) until they reached the adult stage. Considering that the investigated clone did not naturally flower in the field, the maturity of the plant was determined in accordance with hallmarks found within the literature: when plants reached more than 1.5 m height, stems began to wither away and bend and when tuberous roots were completely developed^50^. In the conditions of central Europe, the length of the vegetation period of yacón ranges from 149 to 170 days^50^. In this study, all plants came from the same clone held at the Botanic Garden of the University of Hohenheim, and they were propagated by rhizome cuttings in the form of germinated rhizomes to ensure genetic homogeneity in the studied samples.

Collection of yacón leaves started 15 days after sprouting and continued every 15 days for a period of six months. In each collection, the third pair of leaves (from top to bottom), corresponding to the first well-expanded leaf, was sampled and stored at −70 °C until further use. Collections were always performed at the same hour of the day (between 17:00 and 18:00 h). The development of yacón can be divided into five main growth phases after sprouting (Fig. 2a): leaf development (I), stem ramification (II), crop cover (III), formation of tuberous roots (IV) and flowewing (V)^50^. In each collection, the developmental stage of the plant was recorded. Three biological replicates were collected for each time point.

### Environmental data

For each collection date, climate data from the closest climatological station (Wetterstation Hohenheim) to the cultivation field were obtained. Climate data included temperature (°C), air humidity (%), solar radiation (Wh/m^2^) and rainfall (mm). For data analysis, average (temperature, humidity and rainfall) values for the 15 days prior to each collection were considered. In the case of solar radiation, the accumulated value for the day of collection was considered as previous studies demonstrate that UV-light induces a rapid response in gene expression^45^.

### Metabolite extraction and UHPLC-UV-HRMS analysis

Samples (50 mg/sample – fresh weight) were collected in 2 mL reaction tubes and a 2.8 mm stainless steel bead was added to each of them. After two minutes in liquid nitrogen, samples were ground on a MM300 mixer mill (Retsch GmbH) for 30 s at a frequency of 30 Hz. Homogenized samples were extracted with 70% aqueous ethanol (1 mL) by vortexing for a few seconds followed by ultrasonication at room temperature for 10 min at 40 kHz. After extraction, samples were centrifuged for 5 min at 13,000 rpm and the supernatant was filtered through a 0.2 µm PTFE filter. The extraction process used in this study was based on the protocol of extraction and analysis of plant tissues for metabolomics studies reported by de Vos *et al*.^53^.

Metabolic fingerprinting by UHPLC-UV-HRMS was performed on an Agilent 1290 UHPLC system (Agilent, Germany) coupled to an 80 Hz photodiode array detector (PDA) and a *Q Exactive Plus* (Thermo Scientific, USA) high-resolution mass spectrometer. Chromatographic separation of plant extracts (5 µL) was performed on an Acquity CSH C18 column (1.7 μm, 150 mm × 2.1 mm, Waters, Ireland) using water (0.2% formic acid) as solvent A, acetonitrile (0.2% formic acid) as solvent B (flow rate: 400 μL/min) and the following program: 0–15 min, 3–20% B; 15–40 min, 20–95% B; 40–43 min, 95–3% B; 43–45 min, 3% B. UV detection was performed between 190 and 400 nm.

Mass spectrometry detection was performed in both positive and negative ionization modes using the *Fullscan* (resolution of 70,000 FWHM) and data-dependent MS^2^ (dd-MS^2^, resolution of 17,500 FWHM) methods. Total Ion Current (TIC) chromatograms were obtained over the range of 140–1200 *m/z* using a spray voltage of +4.2 kV and −3.5 kV for the positive and negative ionization modes, respectively. Additional parameters for the mass spectrometer included: capillary temperature, 360 °C; heater temperature, 380 °C; automatic gain control (AGC) target, 1.0e6 (*Fullscan*) and 5.0e4 (dd-MS^2^); maximum inject time, 500 ms (*Fullscan*) and 64 ms (dd-MS^2^); sheath gas flow rate, 60; auxiliary gas flow rate, 20; topN for dd-MS^2^, 5 and an isolation window of 1.5 *m/z*. Nitrogen was used as the drying, nebulizer and fragmentation gas.

### Data preprocessing and multivariate analyses

Chromatographic raw data obtained for each ionization mode (positive and negative) were directly uploaded and processed by MZMine 2.28, where peak detection, peak filtering, chromatogram construction, chromatogram deconvolution, isotopic peak grouping, chromatogram alignment, gap filling, duplicate peaks filter, fragment search and the search for adducts and peak identities were performed^54^. The following MZmine parameters were used for data preprocessing: noise level at 1.0E5; Lorentzian function as peak shape algorithm (resolution of 70,000); minimum peak height at 5.0E5; *m/z* tolerance at 0.002 *m/z* or 5.0 ppm and retention time tolerance of 0.7 min. After MZmine preprocessing, data for each ionization mode were exported as.csv tables with rows representing plant extracts and columns representing ion peak areas associated with a given mass and retention time value. Before statistical analyses, peaks detected in the blank (extraction solvent) and in the final stages of the chromatographic run were removed from the original matrix and the final matrix was scaled by the Pareto method.

Multivariate statistical analyses were performed in the software R 3.0.3 (R Foundation for Statistical Computing, Austria) and SIMCA 13.0.3.0 (Umetrics, Sweden). Initially, a HCAbp was performed using 10,000 bootstrap replicates, the Ward’s method and a Euclidean distance, with the aim of reducing the dataset dimension and recognizing the natural clustering tendency of the samples based on their metabolic fingerprints. To identify the discriminating variables accounting for the observed groups in the HCAbp, a supervised method by OPLS-DA was performed with the same dataset, considering the HCAbp groups as Y variable. Heatmaps were built with all of the identified compounds (see below), using the R package *gplots*, in order to create an overview of the relative accumulation of each metabolite in all samples. Finally, CCA was carried out in the R package *vegan* to correlate the environmental data with the metabolic fingerprints of the samples.

The environmental data sets were processed according to the type of data. Data expressed as percentages (e.g., relative air humidity) were transformed by the arcsine method, while the logarithmic scale was applied to the data not expressed as percentages (e.g., solar radiation, temperature and rainfall) in accordance with the literature^10^.

### Dereplication of plant extracts

Dereplication of plant extracts was performed by molecular networking based on MS/MS spectral similarity, following the online workflow at GNPS^34^. Initially, raw data from each ionization mode (positive and negative) was transformed to nonproprietary.mzXML format using the MSConvert package from the ProteoWizard 3.0.9798 software (Proteowizard Software Foundation, USA) to subsequently upload it to the GNPS platform. Consensus spectra of nearly identical MS/MS spectra were created via MS-Cluster, considering a parent and a fragment ion mass tolerance of 0.02 Da and a minimum of two MS/MS spectra in a consensus to be considered for molecular networking. Networks were created with edges having a cosine score above 0.65 and a minimum of 4 common fragment ions shared by two separate consensus MS/MS spectra in order to be connected by an edge. Edges between two nodes were kept in the network only if both nodes were within the top 10 most similar nodes to each other. Spectral library annotation was performed considering a minimum of 4 common fragment ions, and Cytoscape 3.6.1 was used to display and edit the final structure of the network.

To confirm the compound annotation made by molecular networking, we performed a systematic comparison of retention time, UV, HRMS and MS/MS spectra of experimental data with authentic standards and data from the literature. Accurate mass comparisons were performed relative to the theoretical monoisotopic masses (<3 ppm accuracy) of the secondary metabolites previously reported in the genus *Smallanthus* and in several members of the Asteraceae family (AsterDB), which are freely available at www.asterbiochem.org/asterdb. To identify compounds previously unreported in the genus *Smallanthus*, accurate mass values were used to perform online database searches in the Dictionary of Natural Products (DNP, http://dnp.chemnetbase.com) and in SciFinder Scholar. To confirm peak assignments, the fragmentation patterns of the identified metabolites were proposed based on MS^2^ spectra and the following reference substances: quinic acid, enhydrin, uvedalin and longipilin acetate were used to compare accurate mass measurements and MS^2^ spectra to those of the detected metabolites. Based on the Metabolomics Standard Initiative^55^, three levels of confidence were adopted to report the accuracy of the identifications: level 1 (identified compounds), based upon co-characterization with authentic samples; level 2 (putatively annotated compounds), “based upon physicochemical properties and/or spectral similarity with public/commercial spectral libraries” and level 3 (putatively characterized compound classes), “based upon characteristic physicochemical properties or spectral similarity to known compounds of a chemical class”. In all cases, a minimum of two independent and orthogonal data (e.g., accurate mass and MS^2^ fragmentation patterns or retention time and accurate mass, etc.) were considered. Detailed information regarding all of the identified metabolites is reported in Supplementary Table S1.

### Primer design and sequencing

To investigate the expression patterns of key genes involved in the secondary metabolism of yacón, a species for which scarce information regarding genomic and molecular traits is available, we designed degenerate primers based on homologous sequences from other Asteraceae species (Supplementary Table S2). Genes involved in the biosynthesis of CGAs, flavonoids and STLs, representing the main classes of SMs accumulated in yacón leaves, were investigated. Among the CGA pathways, the enzyme hydroxycinnamoyl CoA: quinate hydroxycinnamoyl transferase (HQT), which catalyzes the biosynthesis of CGA^35^, was selected. Among the flavonoid pathways, the gene coding for the enzyme chalcone synthase (CHS), which catalyzes the conversion of 4-coumaroyl-CoA and malonyl-CoA to naringenin chalcone, was chosen, while for the STL pathways, the gene coding for the enzyme germacrene A oxidase (GAO) was investigated (Fig. 1). GAO catalyzes three consecutive oxidations of germacrene A to yield germacrene A acid, the putative precursor of all STLs in Asteraceae^52^. Actin (ACT) and elongation factor (EF) were selected as housekeeper genes based on literature reports^56^ and their high expression stability values (ACT = 0.03 and EF = 0.015) in the different developmental stages of yacón leaves (Table 1), calculated by the NormFinder algorithm^57^.

**Table 1:** Primer combinations used in PCR and qPCR.

Code	Primer sequence (5′-3′)	Amplicon length (bp)	AT (°C)	Efficiency*	R^2^
Ss_qGAO	F: CGAAAACGGCAACACCACCATT	162	60	92.3	0.998
	R: GCTCGCACCATTGGGAAGTTTC				
Ss_qCHS	F: GCCGACTACCAGCTCACCAAACTC	193	60	87.2	0,999
	R: CCTCATTAGGGCCACGGAACG				
Ss_qHQT	F: GCTACCTTGGGAACGTGGTCTTTACA	194	60	91.2	0.997
	R: GTAACTGGGTCCACGGATTAGAGCC				
Ha_qACT	F: GCCGTGCTTTCTCTTTATGCCAGCGACC	137	60	95.0	0.998
	R: AGCGAGATCAAGACGAAG				
Ha_qEF	F: ACCAAATCAATGAGCCCAAGAGACCCA	131	60	99.6	0.987
	R: TACCGGGCTTGATCACACCAG				
Ss_GAO	F: CARTCGAATGGGCGATTTC	532	54	—	—
	R: CTTTGAACCGTGGCTCCAA				
Ss_CHS	F: AAGCCATYAAAGAATGGGGAAA	729	54	—	—
	R: CCRCCGACTTCTTCCTCATCTC				
Ss_HQT	F: GATATGGCTCGCGGGTTCTC	617	56	—	—
	R: GCATCATGTACTGGTAGCCTGGTC				

*Determined only for qPCR primers; AT: annealing temperature.

For primer design, homologous sequences (complete coding sequences) from several Asteraceae species (Supplementary Table S2) were retrieved from GenBank (www.ncbi.nlm.nih.gov/genbank), aligned in BioEdit^58^ and inspected for conserved regions. Degenerate primers (Table 1) were designed using the software module Primer Select from Lasergene (V7.0.0, DNASTAR) on highly conserved regions and tested on gDNA extracted from yacón leaves following the protocol of Ristaino *et al*.^59^ with the following modification: isopropyl alcohol was used instead of ethanol for DNA precipitation and 40 mg of insoluble polyvinylpyrrolidone (PVP) was added after the nucleus lysis buffer to absorb polyphenols. PCRs were performed in a peqSTAR Thermocycler (Peqlab, Germany) using RNase/DNase-free water and RedTaq MasterMix (Genaxxon Bioscience, Germany). The PCR conditions included 4 min of initial denaturation followed by 38 cycles of 15 s denaturation (96 °C), 15 s annealing (GAO: 54 °C, CHS and HQT: 56 °C), 1 min × kb^−1^ elongation (72 °C) and final elongation for 4 min (72 °C). All PCR products were sequenced by Macrogen (Netherlands), and their identities were confirmed by BLAST searches. After sequencing the target genes on gDNA, the obtained sequences were used as templates to design specific qPCR primers (Table 1) to be used in the gene expression analyses. qPCR primers were tested on yacón cDNA (see below) to confirm the presence of the expected product size and the absence of secondary non-specific bands following the same PCR conditions described above.

### RNA extraction and RT-qPCR

For RNA extraction, six different time points were chosen (0.5, 1.5, 2.5, 3.5, 5.0 and 6.0 months), representing important changes in the yacón metabolome, with one collection for each month. Total RNA from fresh yacón leaves (20 mg) was extracted using the EURx GeneMATRIX Universal RNA Purification kit (Roboklon, Germany), following the manufacturer’s instructions with the following modification: forty milligrams of insoluble PVP was added after the RL buffer to absorb polyphenols. The quantity and purity of the isolated RNA was verified in a BioPhotometer (Eppendorf, Germany). To eliminate residual genomic DNA, the Perfecta Dnase I (Rnase-free) kit (Quanta Biosciences, USA) was used. Samples were confirmed to be free from gDNA by PCR analysis using actin primers. Before cDNA synthesis, the RNA concentration of all samples was adjusted to 50 ng/mL. For qPCR reactions, cDNA synthesis was performed using the RevertAid cDNA Synthesis Kit (Thermo Scientific). The primer used for the first strand synthesis was the VNdT18-Oligonucleotide at a final concentration of 5 µM.

Real time qPCR analyses were performed in a CFX96 Touch™ qPCR System (BioRad GmbH, Germany) and SYBR Green I was used for visualization (SensiFast^TM^ SYBR No-ROX Kit, Bioline). HPLC-purified qPCR primers in a final concentration of 0.25 μM were used. The qPCR program was 2 min at 95 °C for initial denaturation, followed by 45 cycles of 15 s at 95 °C and 15 s at 60 °C. The gene expression analysis was performed using the Bio-Rad CFX Maestro 1.0 software (Bio-Rad Laboratories GmbH). The relative expression of each gene was normalized relative to the youngest stage and to the housekeeper expression of ACT and EF. A melting curve analysis following each RT-qPCR was performed to assess product specificity (Supplementary Fig. S5), and the efficiency of all primers was calculated using four serial dilutions of 1:5 (Supplementary Fig. S6). Gene expression patterns were analyzed in three biological replicates, each with three technical replicates.

## Supplementary information

Supplementary information

Table S1

## Data Availability

Raw LC-MS data and final matrices supporting the metabolomic experiments are available in the MetaboLights repository (Study identifier: MTBLS840).
