# Supplementary material for: Metabolomic and gene expression approaches reveal the developmental and environmental regulation of the secondary metabolism of yacón (Smallanthus sonchifolius, Asteraceae)
Source: Sci Rep. 2019 Sep 11;9:13178. doi: 10.1038/s41598-019-49246-2 (PMC6739394; doi:10.1038/s41598-019-49246-2)
Supplement: Supplementary file 1 — Supplementary information [file 41598_2019_49246_MOESM1_ESM.docx]

# Metabolomic and gene expression approaches reveal the developmental and environmental regulation of the secondary metabolism of yacón (*Smallanthus sonchifolius*, Asteraceae)

Guillermo F. Padilla-González^1^, Maximilian Frey^2^, Javier Gómez-Zeledón^2^, Fernando B. Da Costa*^1^, Otmar Spring^2^.

^1^ AsterBioChem Research Team, Laboratory of Pharmacognosy, School of Pharmaceutical Sciences of Ribeirão Preto, University of São Paulo, Av do café s/n, 14040-903 Ribeirão Preto, SP, Brazil.

^2^ Institute of Botany, University of Hohenheim, Garbenstraße 30, 70599 Stuttgart, Germany.

***** Corresponding author

E-mail: [febcosta@fcfrp.usp.br](mailto:febcosta@fcfrp.usp.br) (FBC)

Tel.: +55 (16) 3315 0661

Supplementary Information


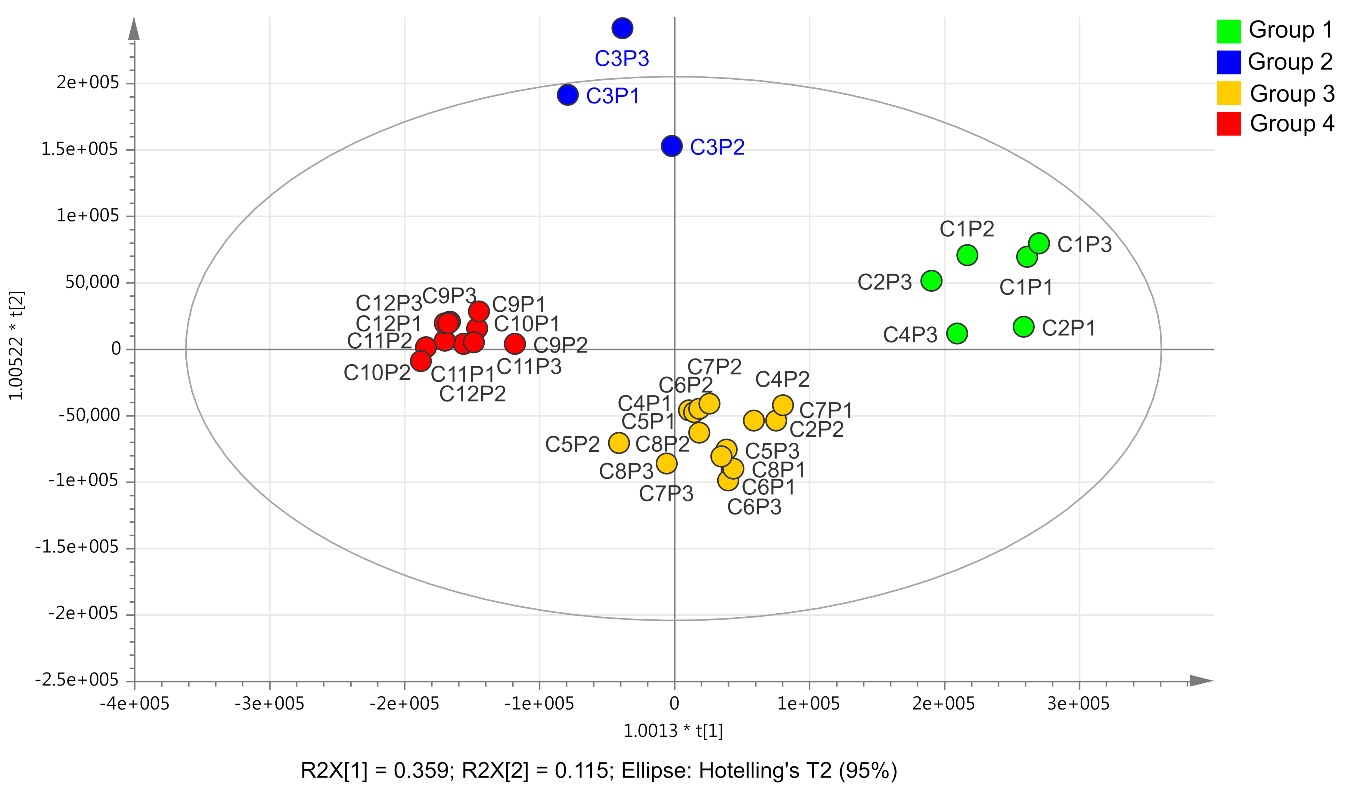


**Fig S1** OPLS-DA scores plot (R^2^=0.93, Q^2^=0.74) based on metabolic profiling by UHPLC-UV-HRMS in positive mode of 36 extracts from *Smallanthus sonchifolius’* leaves collected along the development of the plant (C1 to C12). Samples colored according to their HCA groups (Group 1: 0.5 to 1 month; group 2: 1.5-months; group 3: two to four months and group 4: 4.5 to six months).


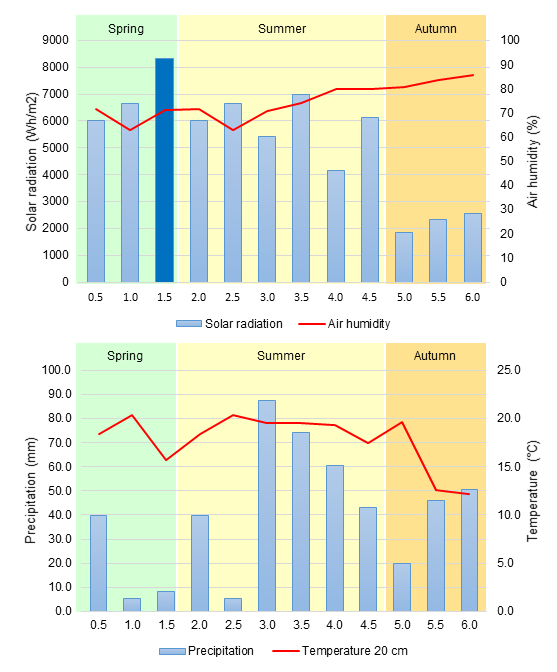


**Fig S2** Climatograms reporting environmental variables from the Hohenheim climate station for the twelve collections of *Smallanthus sonchifolius*’ leaves.


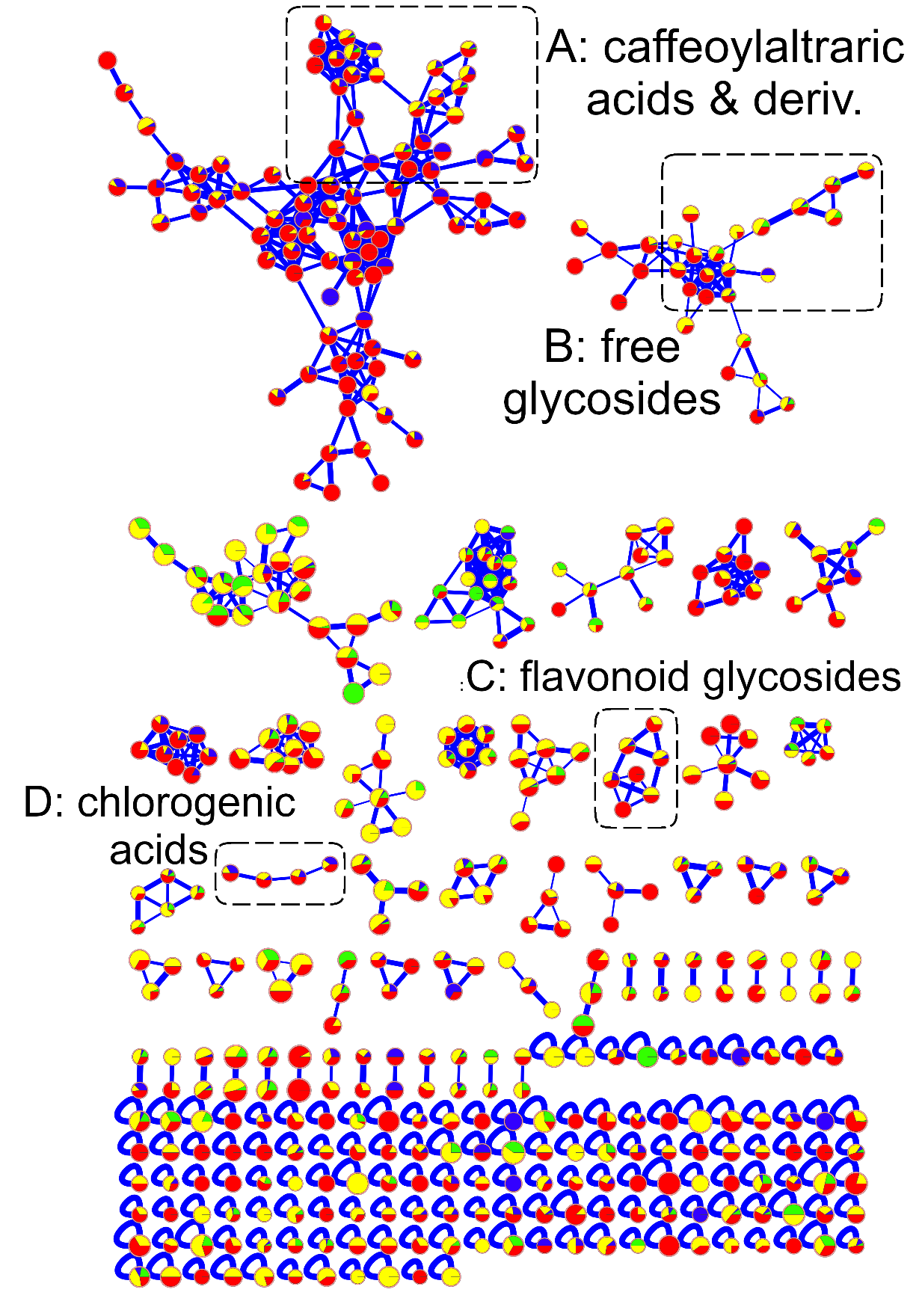


**Fig S3** Molecular networking based on metabolic fingerprinting by UHPLC-HRMS/MS in negative mode of 36 extracts from *Smallanthus sonchifolius’* leaves collected along the development of the plant.

**
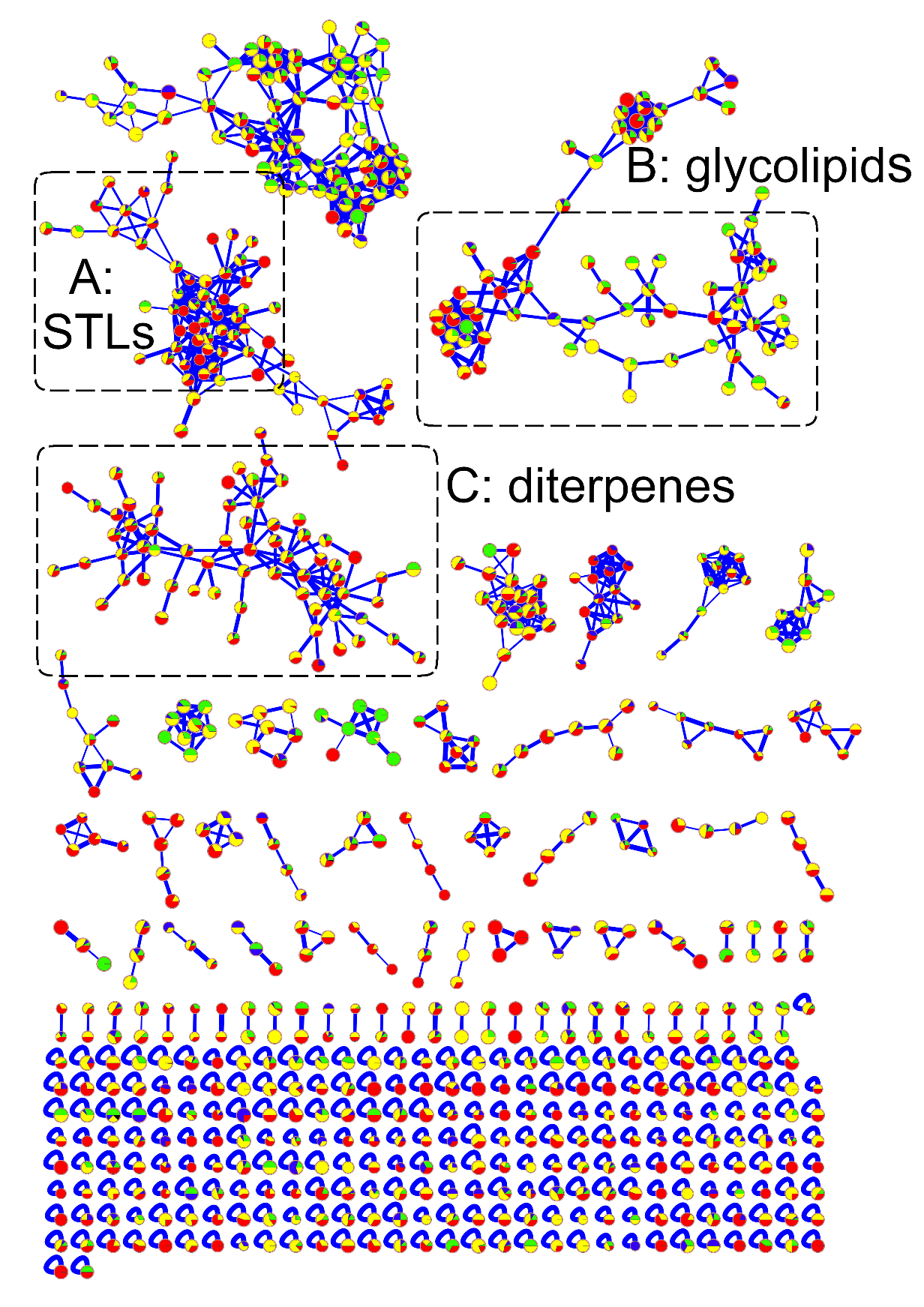
**

**Fig S4** Molecular networking based on metabolic fingerprinting by UHPLC-HRMS/MS in positive mode of 36 extracts from *Smallanthus sonchifolius’* leaves collected along the development of the plant.

**
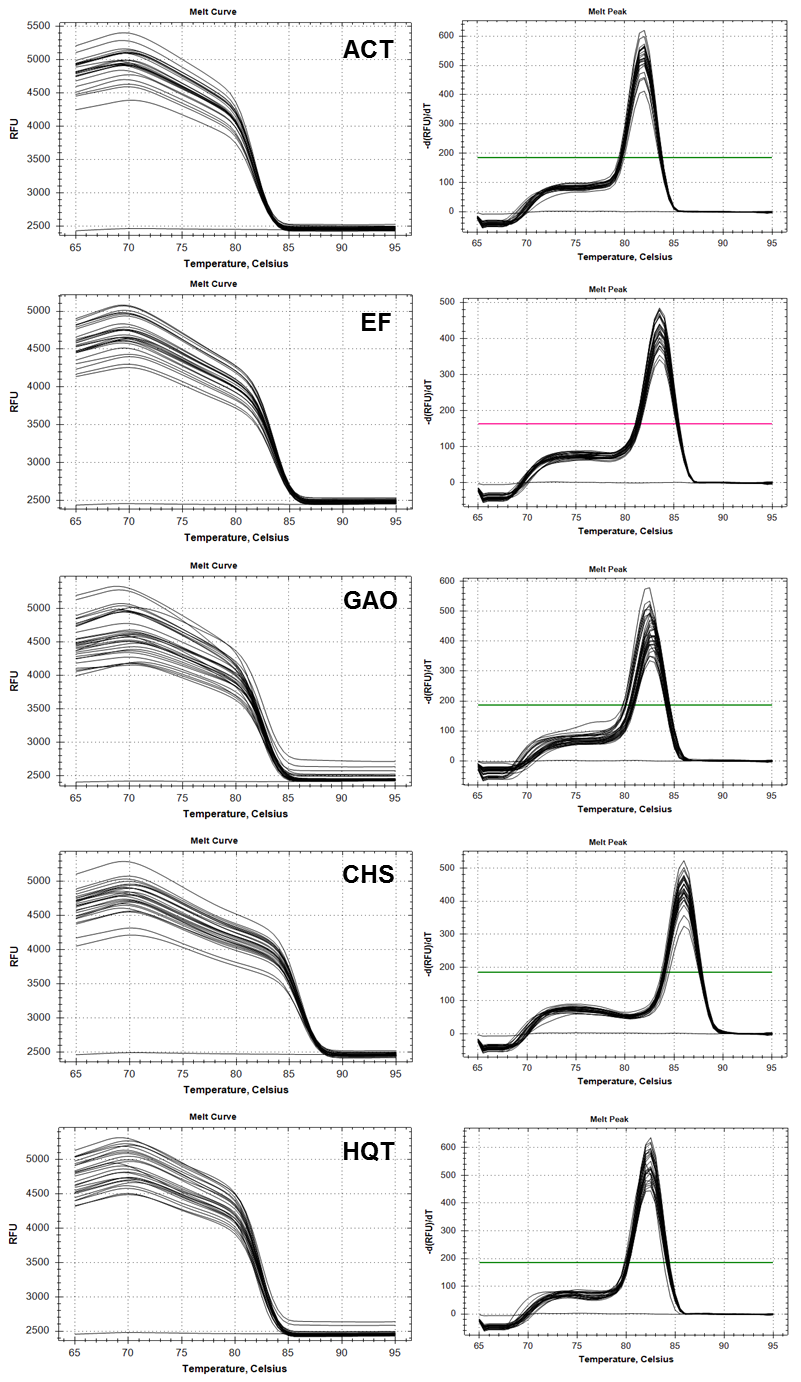
**

**Fig S5** Melt curves of all primers used in the RT-qPCR analyses.


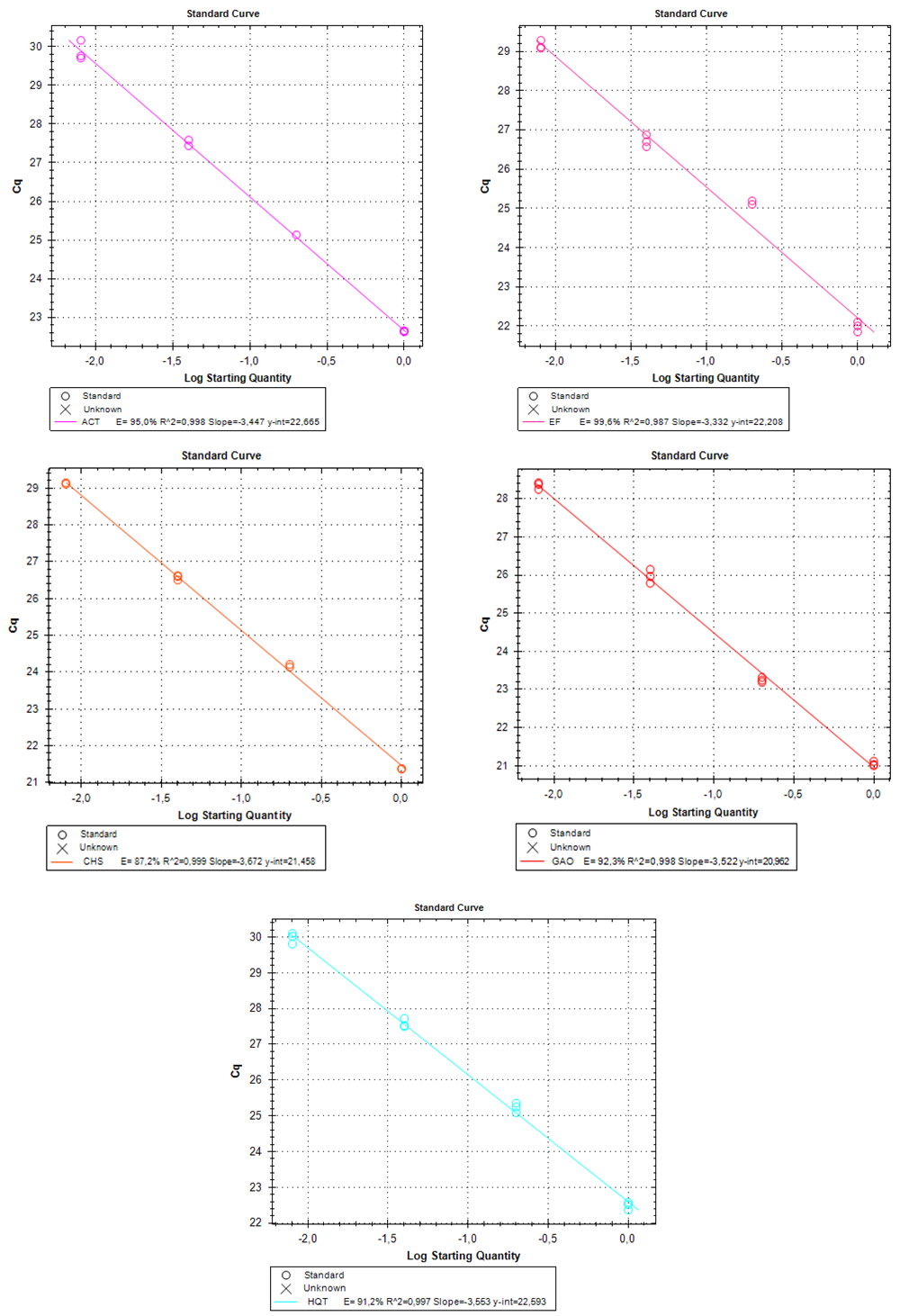


**Fig S6** q-PCR primers efficiency calculated using four serial dilutions of 1:5.

Table S2 Species used to design degenerate primers for GAO, CHS and HQT.

| **Germacrene A oxidase (GAO)** | | **Chalcone synthase (CHS)** | |
| --- | --- | --- | --- |
| **Species** | **Genebank ID** | **Species** | **Genebank ID** |
| *Helianthus annuus* | GU256646.1 | *Helianthus annuus* | KR921882.1 |
| *Lactuca sativa* | GU198171.1 | *Helianthus annuus* | XM_022178725.1 |
| *Tanacetum cinerariifolium* | KC441527.1 | *Helianthus annuus* | XM_022153962.1 |
| *Cichorium intybus* | GU256644.1 | *Rudbeckia hirta* | EF070339.1 |
| *Saussurea costus* | GU256645.1 | *Echinacea pallida* | KY094648.1 |
| *Cynara cardunculus* | KF752448.1 | *Ageratina adenophora* | FJ913888.2 |
| *Barnadesia spinosa* | GU256647.1 | *Dahlia pinnata* | AB591825.1 |
| **Hydroxycinnamoyl-CoA:quinate hydroxycinnamoyltransferase (HQT)** | | *Dahlia pinnata* | AB591826.1 |
| *Cynara cardunculus* | DQ915590.1 | *Carthamus tinctorius* | LC128420.1 |
| *Cynara scolymus* | DQ915589.1 | *Gynura bicolor* | AB550239.1 |
| *Cynara cardunculus* | KU711509.1 | *Eschenbachia blinii* | KJ155749.1 |
| *Cichorium intybus* | KT222893.1 | *Centaurea jacea* | EF112474.1 |
| *Helianthus annuus* | XM_022170642.1 | *Lactuca sativa* | AB525909.1 |
| *Cynara cardunculus* | JF338140.1 |  |  |
